# Complex Regional Pain Syndrome Following CO₂ Laser Labioplasty: A Case Report and Multimodal Interventional Management

**DOI:** 10.7759/cureus.107979

**Published:** 2026-04-29

**Authors:** Joseph A Veraza, Mabel Z Almeida

**Affiliations:** 1 Anesthesiology, Clínica Andes Salud Puerto Montt, Puerto Montt, CHL; 2 Anesthesiology, Hospital Universitario de Caracas, Caracas, VEN; 3 Anesthesiology and Perioperative Medicine, Hospital Universitario de Caracas, Caracas, VEN

**Keywords:** budapest criteria, caudal epidural block, co2 laser surgery, complex regional pain syndrome type 1, ketamine anesthesia, labioplasty, perineal injuries

## Abstract

Complex Regional Pain Syndrome (CRPS) is an uncommon and potentially debilitating neuropathic pain condition that may develop after surgical or iatrogenic nerve injury. Although CRPS predominantly affects the extremities, pelvic and perineal involvement is rare and poorly characterized. We report the case of a 54-year-old woman who developed CRPS following CO₂ laser labioplasty performed as part of a combined gynecologic surgical procedure. The patient presented with progressive neuropathic pain, allodynia, vasomotor and trophic skin changes, and motor impairment predominantly affecting the perineal region and right lower limb. Structural pathology was excluded by imaging. The diagnosis of CRPS was established using the Budapest Criteria. Initial pharmacologic therapy and a selective pudendal nerve block provided limited and transient relief. A multimodal interventional strategy combining repeated caudal epidural blocks, intravenous lidocaine and magnesium sulfate, and epidural preservative-free ketamine resulted in complete pain resolution, reversal of trophic changes, and full functional recovery. This case highlights CRPS as a potential complication of CO₂ laser labioplasty and supports early, mechanism-based multimodal interventional management in refractory cases, particularly when central sensitization is suspected.

## Introduction

Complex Regional Pain Syndrome (CRPS) is a chronic neuropathic pain disorder characterized by persistent regional pain disproportionate to the inciting event and accompanied by sensory, vasomotor, sudomotor, and motor or trophic abnormalities. The condition is thought to arise from a complex interaction of peripheral nerve injury, central sensitization, autonomic dysregulation, and neuroinflammatory processes [1-3]. Diagnosis is clinical and relies on the validated Budapest Criteria, which require the presence of specific symptom and sign categories to improve diagnostic specificity [4]. Although CRPS most commonly affects the upper and lower extremities, atypical presentations involving the pelvic or perineal regions have been described, often following surgical or iatrogenic nerve injury [5].

CO₂ laser labioplasty is generally considered a safe procedure; however, the close anatomical relationship between laser-treated tissues and sensory nerve branches, including the pudendal nerve, may predispose to thermal nerve injury. We describe an unusual case of CRPS following CO₂ laser labioplasty and present a successful multimodal interventional management strategy.

## Case presentation

A 54-year-old woman (weight 58 kg, height 162 cm) with a history of well-controlled arterial hypertension underwent an abdominal hysterectomy combined with CO₂ laser labiaplasty for symptomatic labial hypertrophy. Neuraxial anesthesia was administered using a combined spinal-epidural technique. Reduction labiaplasty was performed using a CO₂ laser in continuous wave (CW) mode, with tissue excision carried out using a focused beam at 10-15 W. Hemostasis and surgical edge coagulation were achieved using a defocused beam at 2.5-3.0 W. The immediate postoperative course was uneventful, and the patient was discharged pain-free.

**Figure 1:**
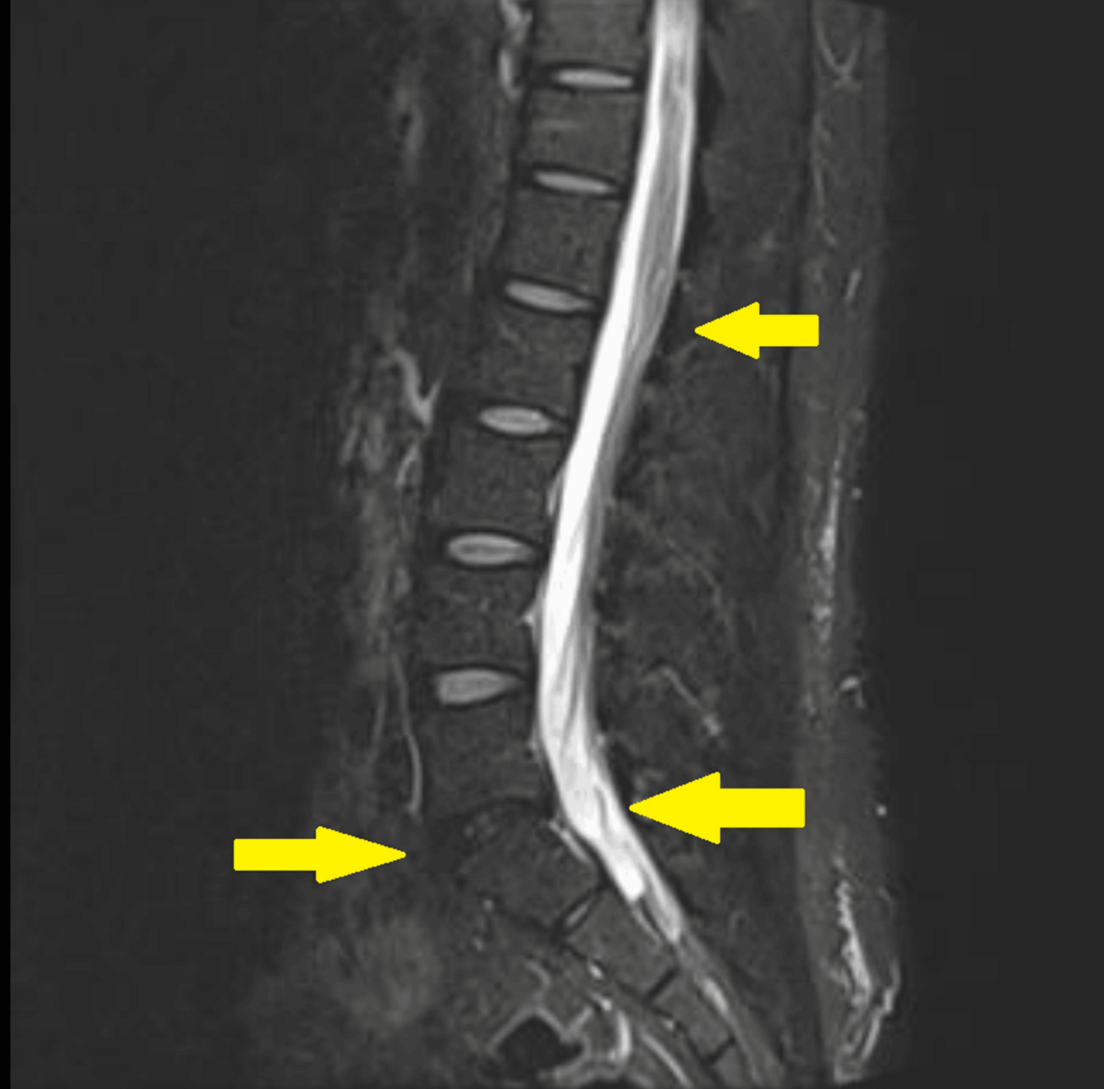
Normal MRI of the lumbosacral spine Sagittal T2-weighted magnetic resonance imaging (MRI) of the lumbosacral spine. The yellow arrows indicate normal anatomical structures, including the spinal canal, cauda equina, and intervertebral discs, with no evidence of compressive or inflammatory neuraxial pathology. This normal finding was crucial as a diagnosis of exclusion to rule out structural causes for the patient's symptoms, thereby supporting the clinical diagnosis of Complex Regional Pain Syndrome (CRPS).

On postoperative day 5, she developed burning perineal pain associated with hyperesthesia and allodynia (Numeric Rating Scale [NRS] 5/10). Initial pharmacologic management included oral paracetamol 1 g every 6 hours and ibuprofen 600 mg every 8 hours; however, these interventions failed to provide adequate analgesia, with persistent and progressive symptomatology. By postoperative day 7, pain intensity increased (NRS 7/10) and was accompanied by paresthesia in the lower limbs and difficulty with defecation. Magnetic resonance imaging of the lumbosacral spine excluded compressive or inflammatory neuraxial pathology (Figure 1).

Despite multimodal intravenous analgesia with ketorolac 90 mg plus dexamethasone 8 mg and metamizole 5 g in a drip of 500 ml during 24 hours and transdermal opioid therapy (buprenorphine 10 mcg/h), symptoms progressed. By postoperative day 20, the patient developed marked motor weakness predominantly affecting the right lower limb, violaceous discoloration of the vaginal region, severe perineal and thigh allodynia, gait impairment, and inability to tolerate clothing contact. A selective ultrasound-guided pudendal nerve block resulted in transient symptom improvement. The presence of persistent disproportionate pain, sensory abnormalities, vasomotor changes, sudomotor dysfunction, motor impairment, and trophic alterations fulfilled the Budapest diagnostic criteria for CRPS. Psychiatric evaluation revealed significant psychological distress related to loss of functional autonomy.

**Figure 2:**
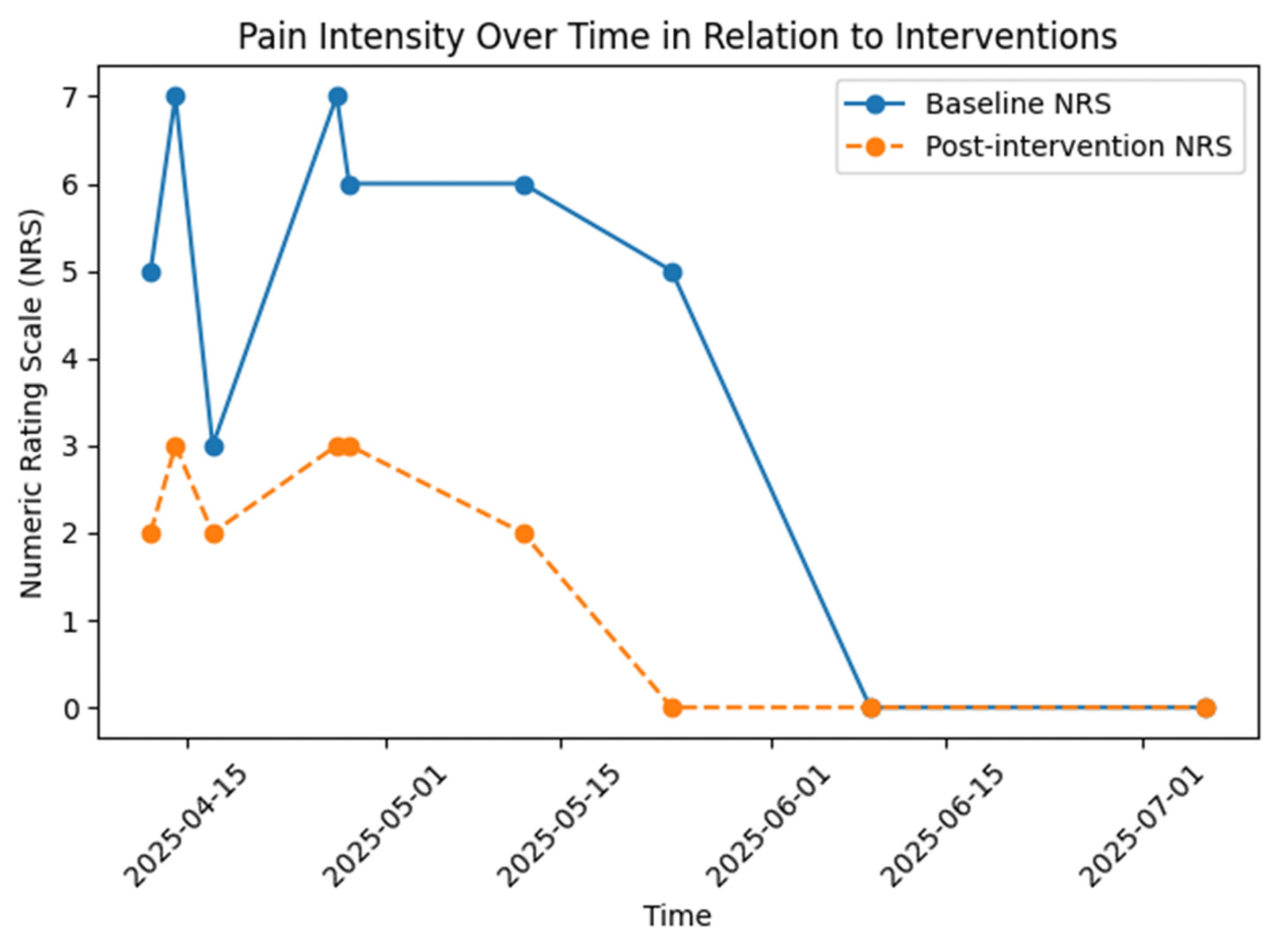
Temporal Evolution of Pain Intensity in Relation to Interventions The graph illustrates the patient's reported pain severity over the clinical course, measured by the Numeric Rating Scale (NRS), in relation to the timeline of pharmacological and interventional treatments. Key milestones include the onset of severe neuropathic pain, the diagnosis of Complex Regional Pain Syndrome (CRPS), and the progressive reduction in pain scores following targeted multimodal therapies, culminating in complete symptom resolution. NRS: Numeric Rating Scale; CRPS: Complex Regional Pain Syndrome; IV: Intravenous.

A multimodal interventional pain management strategy was initiated. Repeated fluoroscopically guided caudal epidural blocks were performed using bupivacaine 0.125% (20 mL), combined with intravenous magnesium sulfate (2 g over 1 hour) and intravenous lidocaine (1.5 mg/kg bolus followed by 2 mg/kg/h infusion for 2 hours). Due to partial improvement, preservative-free racemic ketamine (50 mg) was added to the caudal epidural injectate. Following four treatment sessions over two consecutive weeks, the patient achieved complete pain resolution (NRS 0), reversal of trophic skin changes, and progressive recovery of muscle strength. She resumed independent ambulation and returned to work. At final follow-up, she remained asymptomatic without functional limitations. The chronological clinical course, therapeutic interventions, and corresponding pain intensity scores are summarized in Table 1, while the temporal evolution of pain severity in relation to key interventions is illustrated in Figure 2.

**Table 1:** Chronological clinical course, interventions, and pain intensity Summary of the chronological clinical course, therapeutic interventions, and corresponding pain intensity. Pain severity was evaluated using the Numeric Rating Scale (NRS; 0 = no pain, 10 = worst pain imaginable) at baseline and following each specific therapeutic intervention. NRS: Numeric Rating Scale; NSAIDs: Non-steroidal anti-inflammatory drugs; IV: Intravenous; CRPS: Complex Regional Pain Syndrome.

Date	Clinical status	Intervention	NRS (baseline)	NRS (post-intervention)
Apr 12, 2025	Initial perineal burning pain and allodynia	Oral NSAIDs + paracetamol	5	2
Apr 14, 2025	Hospital readmission with severe neuropathic pain	IV multimodal analgesia with paracetamol 1 g every 6 hours + drip of ketorolac 90 mg/dexamethasone 8 mg/metamizole 5 g during 24h + methadone rescue (3 mg SOS)	7	3
Apr 17, 2025	Discharge with partial improvement	Buprenorphine patch (10 mcg/h) + oral analgesics paracetamol 1 g every 6 hours + ibuprofen 600 mg every 8 hours	3	2
Apr 27, 2025	Progressive motor weakness and trophic changes	Neurology evaluation + pregabalin 75 mg	7	3
Apr 28, 2025	Selective pudendal nerve block5	Ultrasound-guided pudendal nerve block with bupivacaine 0.25% 10 ml	6	3
May 12, 2025	Diagnosis of CRPS (Budapest criteria)	Caudal block + IV lidocaine (1.5 mg (7 mg/kg/bolus + infusion 2 mg/kg/h) and magnesium 2 g during 2 hours	6	2
May 24, 2025	Persistent pain with partial improvement	Caudal block + IV therapy + epidural ketamine 50 mg	5	0
Jun 09, 2025	Complete resolution of pain	Maintenance therapy + physiotherapy	0	0
Jul 06, 2025	Asymptomatic, full recovery	Discharged from pain clinic	0	0

## Discussion

This case describes an uncommon presentation of CRPS involving the perineal and pelvic region following CO₂ laser labiaplasty. While CRPS is classically associated with limb trauma, this report highlights that iatrogenic nerve injury in anatomically complex regions may similarly trigger maladaptive pain mechanisms.

The Budapest Criteria represent the most specific clinical framework for CRPS diagnosis and were fully met in this patient [4]. The presence of sensory disturbances, vasomotor and sudomotor abnormalities, motor dysfunction, and trophic changes, combined with the exclusion of structural pathology, supports the diagnosis and differentiates CRPS from isolated postoperative neuropathy.

In accordance with the bio-psycho-social model of CRPS, a structured psychological evaluation was integrated into the patient’s management. Psychological distress was assessed using validated instruments, including the Generalized Anxiety Disorder 7-item (GAD-7) scale and the Pain Catastrophizing Scale (PCS), which revealed moderate anxiety and significant pain-related rumination. To address these factors, the patient was referred for targeted cognitive behavioral therapy focused on pain coping and desensitization strategies. In parallel, pharmacologic management was optimized with a dual-acting agent (duloxetine), targeting both neuropathic pain and associated mood disturbances. This integrated approach allowed simultaneous management of central sensitization and psychological contributors, consistent with current CRPS treatment paradigms.

The transient improvement observed after a selective pudendal nerve block initially suggested a localized neuropathic process. However, several features argued against isolated pudendal neuralgia and supported a diagnosis of CRPS type II. Notably, the presence of vasomotor changes (violaceous discoloration), sudomotor dysfunction, trophic alterations, and progressive motor impairment extending beyond the pudendal nerve distribution are not characteristic of a focal nerve entrapment. In addition, the spread of symptoms to the lower extremity and the development of functional impairment further support a centralized pain process rather than a purely peripheral neuropathy. These findings, in conjunction with fulfillment of the Budapest Criteria and exclusion of structural pathology, were decisive in establishing the diagnosis of CRPS.

CRPS pathophysiology involves peripheral nerve injury leading to neurogenic inflammation, central sensitization, and autonomic dysfunction [1-3]. Thermal injury from CO₂ laser energy may have affected branches of the pudendal or genitofemoral nerves, initiating aberrant nociceptive signaling and sympathetic dysregulation. Pelvic CRPS presentations are likely underrecognized due to atypical distribution and diagnostic delay.

The progression from a localized perineal pain syndrome to lower limb sensory and motor involvement raises important considerations regarding the underlying neurophysiological mechanisms. While CRPS is classically described as a regionally confined condition, contiguous spread beyond the initial site has been reported. In this case, structural causes were excluded by lumbosacral imaging, supporting a functional neurogenic process. One plausible explanation involves central sensitization and spinal cord-level neuronal plasticity, whereby persistent nociceptive input from the pudendal nerve may lead to cross-excitation of adjacent neural segments, resulting in symptoms extending to the lower extremities. Although a functional (psychogenic) component cannot be entirely excluded, the presence of objective sensory disturbances, trophic changes, and response to interventional therapies supports an organic neuropathic mechanism.

Early multidisciplinary management is recommended in CRPS to prevent chronicity and irreversible functional impairment [6]. Pharmacologic therapies alone often provide limited benefit in severe cases [6,7]. In this patient, a mechanism-based multimodal interventional approach was employed. Caudal epidural blockade likely reduced afferent nociceptive input and sympathetic outflow, while systemic lidocaine and magnesium sulfate contributed sodium channel blockade and NMDA receptor antagonism.

The administration of intravenous lidocaine and magnesium sulfate was based on their established roles as adjuncts in neuropathic pain and central sensitization. Lidocaine was administered as a bolus followed by continuous infusion within commonly reported safe dosing ranges, aiming to achieve analgesic effects through sodium channel blockade without reaching toxic plasma concentrations. Although plasma lidocaine levels were not routinely measured, patients were closely monitored for signs of systemic toxicity, and no adverse effects were observed. Magnesium sulfate was administered as an NMDA receptor antagonist to enhance modulation of central sensitization, consistent with protocols described in perioperative and chronic pain management literature. These pharmacologic strategies were selected to target both peripheral and central mechanisms of CRPS while maintaining an acceptable safety profile.

The addition of neuraxial ketamine may have further attenuated central sensitization, consistent with evidence supporting ketamine’s role in refractory CRPS [8].

Although the multimodal approach limits the ability to isolate the individual contribution of each intervention, the temporal association between the addition of neuraxial ketamine and the patient’s subsequent complete pain resolution suggests a central role for NMDA receptor antagonism in this case. Lidocaine, magnesium sulfate, and caudal epidural blockade were administered concurrently and may have contributed to the modulation of nociceptive input; however, the definitive turning point in pain trajectory occurred following the introduction of ketamine. This observation is consistent with existing evidence supporting ketamine’s efficacy in refractory CRPS, particularly in the context of established central sensitization [8].

Our findings align with emerging literature suggesting that CRPS can occur in atypical anatomical regions following minimally invasive gynecological or pelvic surgeries. For instance, pelvic and perineal CRPS has been previously described as a rare but severe complication following procedures such as transvaginal surgeries and pelvic floor repairs [9]. In these instances, much like in our patient, the rich innervation of the pelvic region and the potential for indirect thermal or traction injury play a pivotal role in initiating the neuropathic cascade. This reinforces the need for early multimodal intervention before central sensitization becomes entrenched [10, 11].

## Conclusions

CRPS should be considered in patients presenting with disproportionate perineal pain and autonomic or trophic changes following CO₂ laser labioplasty. Early diagnosis and a structured, multimodal interventional strategy targeting peripheral and central sensitization are crucial. In this specific case, the prompt initiation of caudal epidural blocks combined with intravenous magnesium and lidocaine, followed by epidural ketamine, successfully reversed all trophic changes and achieved a complete, lasting resolution of pain, allowing the patient to return fully to her baseline functional status.
